# Characterization and Comparison of Convergence Among *Cephalotus follicularis* Pitcher Plant-Associated Communities With Those of *Nepenthes* and *Sarracenia* Found Worldwide

**DOI:** 10.3389/fpls.2022.887635

**Published:** 2022-06-06

**Authors:** Leonora S. Bittleston, Elizabeth L. Benson, Jessica R. Bernardin, Naomi E. Pierce

**Affiliations:** ^1^Department of Biological Sciences, Boise State University, Boise, ID, United States; ^2^Department of Organismic and Evolutionary Biology, Harvard University, Cambridge, MA, United States

**Keywords:** carnivorous plant, bacteria, eukaryote, convergent evolution, microbe, microbiome

## Abstract

The Albany pitcher plant, *Cephalotus follicularis*, has evolved cup-shaped leaves and a carnivorous habit completely independently from other lineages of pitcher plants. It is the only species in the family Cephalotaceae and is restricted to a small region of Western Australia. Here, we used metabarcoding to characterize the bacterial and eukaryotic communities living in *C. follicularis* pitchers at two different sites. Bacterial and eukaryotic communities were correlated in both richness and composition; however, the factors associated with richness were not the same across bacteria and eukaryotes, with bacterial richness differing with fluid color, and eukaryotic richness differing with the concentration of DNA extracted from the fluid, a measure roughly related to biomass. For turnover in composition, the variation in both bacterial and eukaryotic communities primarily differed with fluid acidity, fluid color, and sampling site. We compared *C. follicularis*-associated community diversity with that of Australian *Nepenthes mirabilis*, as well as a global comparison of Southeast Asian *Nepenthes* and North American *Sarracenia*. Our results showed similarity in richness with communities from other pitcher plants, and specific bacterial taxa shared among all three independent lineages of pitcher plants. Overall, we saw convergence in richness and particular clades colonizing pitcher plants around the world, suggesting that these highly specialized habitats select for certain numbers and types of inhabitants.

## Introduction

Carnivorous pitcher plants are a clear example of convergent evolution, where the same form and function has evolved independently across three different lineages (Albert et al., 1992). These “true” pitcher plants have pitcher-shaped, single leaves containing small ecosystems with complex communities of arthropods and microbes (Adlassnig et al., 2011). In recent years, there has been a growing recognition of the importance of plant-associated communities beyond just the organisms that are clearly symbionts or pathogens (Bell et al., 2019; Leveau, 2019). Pitcher plants provide an opportunity to examine how hosts with similar morphology and life-history traits that have evolved independently in dramatically different geographic locations, can nevertheless attract and cultivate similar communities within their structures. Pitcher plant-associated communities from the genera *Sarracenia* (family Sarraceniaceae) and *Nepenthes* (family Nepenthaceae) have now been characterized across numerous studies (Peterson et al., 2008; Koopman and Carstens, 2011; Gray, 2012; Gray et al., 2012; Kanokratana et al., 2016; Bittleston et al., 2018; Grothjan and Young, 2019; Gilbert et al., 2020a,b). Many of the same bacterial and eukaryotic taxonomic groups are common in both *Sarracenia* and *Nepenthes* communities and their metagenomes show enrichment in specific degradation genes (Bittleston et al., 2018), suggesting that convergent interactions (Bittleston et al., 2016b) may exist among these convergently evolved plants and the organisms living in their pitchers. However, there is very little known about the communities associated with natural populations of the third pitcher plant lineage in the genus *Cephalotus*, and its bacterial microbiome has never been characterized.

**Figure 1:**
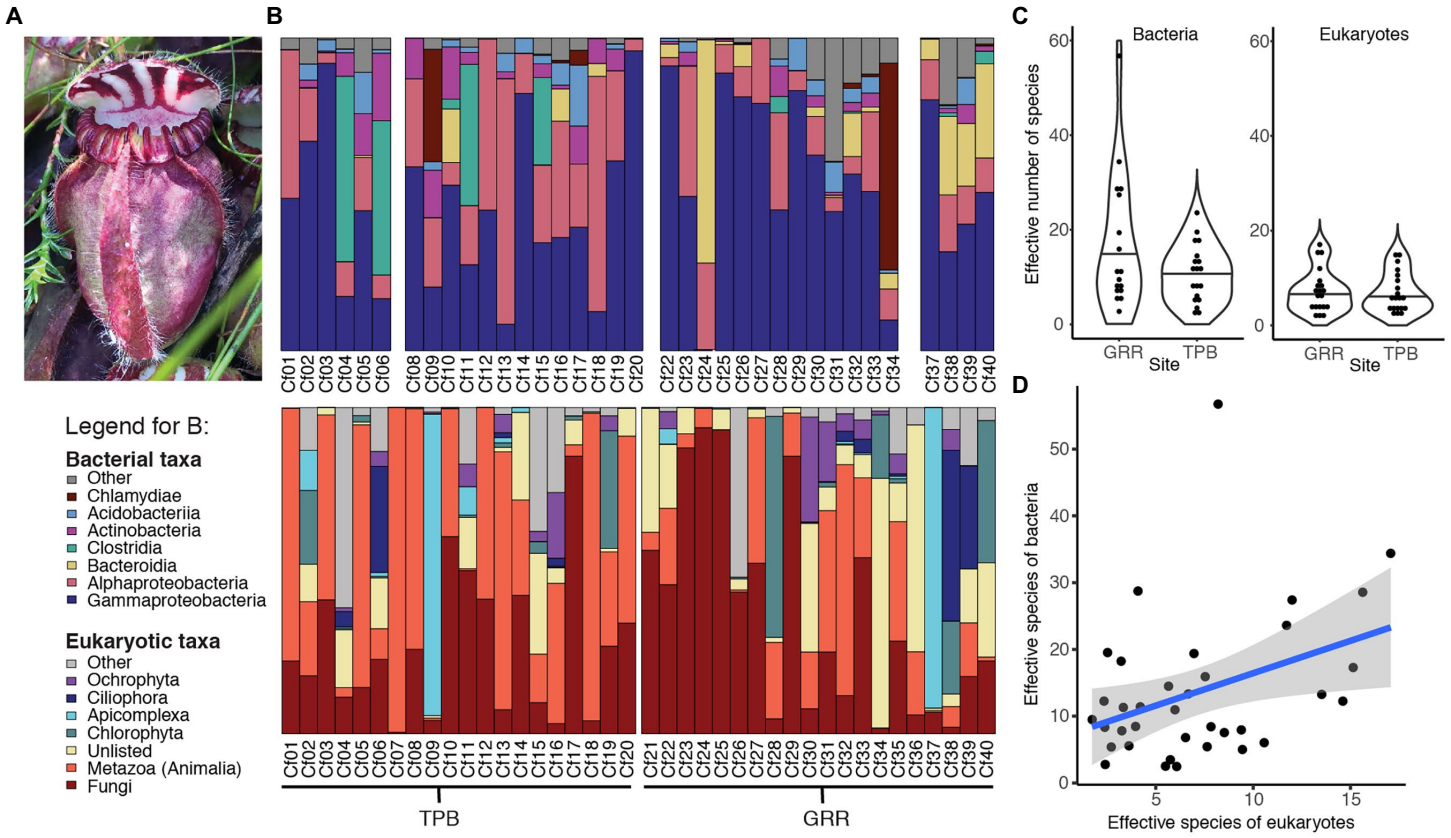
Characterizing *Cephalotus follicularis*-associated communities. **(A)** Image of *C. follicularis* pitcher in its natural habitat (Photo credit: L.S. Bittleston). **(B)** Relative abundances of the most common bacterial (top) and eukaryotic (bottom) taxa across our samples. The first 20 were from site Two Peoples Bay (TPB) and the second set from Gull Rock Road (GRR). Some bacterial samples did not sequence well (Cf07, 21, 35, and 36); those columns are left blank. **(C)** Richness, measured as effective number of species [the exponential of Shannon diversity of the amplicon sequence variants (ASVs)] by site for bacteria (left) and eukaryotes (right). **(D)** Bacterial and eukaryotic richness (effective numbers of species) are correlated.

The Albany pitcher plant, *C. follicularis* (Figure 1A), is the only genus and species in the family Cephalotaceae (Conran, 2004). It is endemic to the coastal region of South-Western Australia near the town of Albany, and few studies have examined the plant and its associated organisms in its natural habitat (Parkes and Hallam, 1984; Clarke, 1988; Yeates, 1992; Pavlovic, 2011; Lymbery et al., 2016; Just et al., 2019). Despite their small native range, the unusual *C. follicularis* plants are prized by collectors and have been cultivated and sold around the world (Conran, 2004). The plants grow in wet, peat-soil swamps often in seepage areas (Conran, 2004) and have been shown to get about 26% of their nitrogen from captured prey (Schulze et al., 1997). Recently, the genome of *C. follicularis* was sequenced, and transcriptomic analysis of carnivorous vs. non-carnivorous leaves identified genetic changes associated with carnivory (Fukushima et al., 2017). This diminutive and charismatic plant is listed as Vulnerable by the IUCN, due to population reduction from habitat loss and overcollection, highlighting the necessity for a better understanding of its ecology.

Carnivorous pitcher plants trap and digest insects as a strategy for gaining nitrogen, phosphorus and other nutrients that are lacking in the generally water-logged soils where they grow. Despite their consumption of insect prey, a living community of arthropods, protozoa, algae, fungi, and bacteria thrive in the aquatic pitcher pools (Kitching, 2000). Pitcher microbiomes likely assist in degradation of prey and support plant nutrient access (Butler et al., 2008; Luciano and Newell, 2017; Bittleston et al., 2018). In *Nepenthes* and *Sarracenia* species, pitcher-associated communities have been found to vary by host species and pitcher fluid pH and volume, among other characteristics (Kanokratana et al., 2016; Bittleston et al., 2018; Gilbert et al., 2020b). Pitcher plants, despite being unusual within the plant world, are an emerging model system for community ecology because their pitchers contain small, naturally replicated microcosms that assemble anew each time a pitcher opens (Srivastava et al., 2004; Peterson et al., 2008; Miller et al., 2018; Ellison and Gotelli, 2021).

The extreme geographic isolation of *C. follicularis* from other pitcher plants may lead to it hosting fewer metazoan inhabitants (inquilines). A study of aquatic organisms, ranging from bacteria to fish, found higher dispersal limitation in larger organisms (De Bie et al., 2012). Beaver (1985) and later Clarke (1988) hypothesized that metazoan food web complexity in *Nepenthes* pitcher plants had geographical underpinnings, related to factors such as landmass size, degree of spatial and temporal isolation, the size of the local species pool capable of colonizing pitchers and the number of *Nepenthes* species present. One of the few known pitcher inhabitants of *C. follicularis*, and the only insect, is the larvae of the fly *Badisis ambulans*. This inquiline has never been found outside of *C. follicularis* pitchers and its interaction with the plant may be mutualistic or parasitic (Yeates, 1992; Lymbery et al., 2016). The thesis of Sally A. Clarke examined *C. follicularis*-associated protists in depth, but did not list many metazoans.

To the best of our knowledge, this is the first metabarcoding study characterizing the entire bacterial and eukaryotic communities of *C. follicularis* pitcher pools and comparing them with those of other pitcher plant species. Here, we aim to: (1) characterize the bacteria and eukaryotes associated with *C. follicularis* pitchers using DNA metabarcoding, and to assess how they are affected by site and host characteristics and (2) investigate possible convergence in community richness and shared taxa due to the very distinctive and similar life form among pitcher plants. We ask: What organisms live in *C. follicularis* pitchers? How do growing site and characteristics of the pitcher fluid impact community composition and structure? Do different factors affect bacterial and eukaryotic communities? Are *C. follicularis* pitcher communities similar to those found in Australian *Nepenthes*? Do we see convergent interactions such as those found in *Sarracenia* and *Nepenthes*?

## Materials and Methods

### Sampling in Australia

In January 2016, we collected fluid samples from 40 healthy *C. follicularis* pitchers, split evenly between two sites roughly 20 km apart near Albany, Western Australia designated Gull Rock Road (GRR) and Two Peoples Bay (TPB; GPS coordinates are not listed here to protect the vulnerable plants but are available from the authors upon request). We haphazardly selected 20 healthy pitchers on different plants at each site that had no visible damage. Although it is difficult to gauge pitcher time-since-opening in the field without marking unopened pitchers and returning over time, we aimed to select pitchers of different ages to get a full representation of the existing diversity. The entire pitcher fluid from each pitcher was collected using a sterile pipette for each sample and was transferred to sterile tubes. Fluid color was noted qualitatively by a single researcher and total volume was recorded before removing an aliquot of fluid for pH measurement and sequencing. We measured the pH of the samples with Macherey-Nagel pH-Fix 4.5–10.0 and BDH VWR Analytical pH-Test 0.0–6.0 indicator strips. We preserved samples with a 1:1 volume of cetyl trimethylammonium bromide (CTAB) buffer at room temperature before transporting them to the laboratory and freezing them until DNA extraction. In addition to these samples of *C. follicularis*, 21 *N. mirabilis* pitchers were sampled near Bramston Beach, Queensland, Australia in May 2016 following the same protocol as the *C. follicularis* sample collection.

### DNA Extraction and 16S- and 18S-Amplicon Next-Generation Sequencing

We extracted DNA from sample fluids using a phenol-chloroform bead-beating extraction method (Sambrook et al., 2001; Bittleston et al., 2018). DNA extracts were quantified with a Qubit fluorometer. DNA amplification and Illumina MiSeq next-generation sequencing were performed at Argonne National Laboratory’s Environmental Sample Preparation and Sequencing Facility (ESPSF). We used the Earth Microbiome Project’s original barcoded 16S (515F-806R, V4 region) and 18S (1391f-1510r, V9 region) primers that were adapted for the Illumina MiSeq (Amaral-Zettler et al., 2009; Stoeck et al., 2010; Caporaso et al., 2012). Each 25 μl PCR reaction contained 9.5 μl of MO BIO PCR Water (Certified DNA-Free), 12.5 μl of QuantaBio’s AccuStart II PCR ToughMix (2x concentration, 1x final), 1 μl Golay barcode tagged Forward Primer (5 μM concentration, 200 pM final), 1 μl Reverse Primer (5 μM concentration, 200 pM final), and 1 μl of template DNA. The conditions for PCR were: 94°C for 3 min, with 35 cycles at 94°C for 45 s, 50°C for 60 s, and 72°C for 90 s; with a final extension of 10 min at 72°C. Amplicons were quantified using PicoGreen (Invitrogen) and a plate reader (Infinite 200 PRO, Tecan) before being pooled in equimolar amounts. This pool was then cleaned with AMPure XP Beads (Beckman Coulter), and quantified using a fluorometer (Qubit, Invitrogen). After quantification, the molarity of the pool was determined and it was diluted down to 2 nM, denatured, and then diluted to a final concentration of 6.75 pM with a 10% PhiX spike for sequencing on the Illumina MiSeq. The 16S and 18S amplicons were sequenced on separate 151 × 12 × 151 bp MiSeq runs.

Amplicon sequence data for the Australian samples (*C. follicularis* and *N. mirabilis*) have been deposited in the Sequence Read Archive NCBI BioProject PRJNA810039.

### Inclusion of Data From *Nepenthes*, *Sarracenia*, and Surrounding Habitats

In order to compare our samples from *C. follicularis* and *N. mirabilis* pitchers with other pitcher plant and environmental communities, we used existing data from Bittleston et al., 2018 (sequence data are available from BioProject PRJNA448553). The comparison samples are described in detail in Bittleston et al., 2018 and were collected, transported, extracted and sequenced in very similar ways.

The environmental samples represent the habitats were the *Nepenthes* and *Sarracenia* plants were growing, e.g., the bog water or soil directly next to the plants. Unfortunately, due to the restrictions of our Australian permits, we were not able to collect soil or bog water samples at our Australian sites. Our comparison with soil and bog water is limited to environmental samples collected from the direct surroundings of *Nepenthes* and *Sarracenia* in Southeast Asia and North America.

### Sequencing Quality Control and Statistical Analyses

To be as fully comparable as possible, all samples (those collected in Australia in 2016 as well as those that had been previously collected elsewhere and sequenced) were analyzed together in the same way. We used QIIME2 versions 2018.4 and 2019.10 (Bolyen et al. 2019) on the Boise State computing cluster for quality control, Amplicon Sequence Variant (ASV) generation, taxonomic assignment, and phylogenetic trees. We denoised sequences and generated ASVs with the DADA2 (Callahan et al., 2016) plugin, and 16S taxonomy assignment with the classify-sklearn Naive Bayes classifier (Pedregosa et al., 2011), and a pre-trained classifier made with the Greengenes database, version 13_8 (McDonald et al., 2012; Bokulich et al., 2018). For 18S taxonomy assignment, we used the classify-consensus-vsearch method and the SILVA 128 database. For both loci, we used the QIIME2 SEPP plugin (Janssen et al., 2018) to build phylogenies. All downstream analyses were done in R (version 4.0.2, R Core Team, 2020).

We removed all sequences assigned as chloroplasts from the 16S data, and bacteria and archaea from the 18S data. To minimize misassigned sequences and to focus on organisms most likely to be associated with pitchers, we removed all observations with fewer than 10 sequences and samples with fewer than 1,000 sequences. We focused on *C. follicularis* communities first, creating a subset of just these samples and rarefying at 4,039 sequences for 16S and 2,704 for 18S. We ran generalized linear models (GLMs) using a Gamma distribution (log link function) to examine the association of bacterial and eukaryotic richness (measured as Effective Number of Species, a Hill number that is the exponential of the Shannon index; Jost, 2007; Chao et al., 2014) with site, DNA concentration, and pitcher fluid pH, volume, and color. We chose to use GLMs in order to measure all of our variables together to control for their effects on each other and to avoid transforming non-normally distributed data. We made sure that our data met the assumptions for a Gamma GLM (see R code in the Dataverse repository). For beta diversity analyses, we used unweighted UniFrac, a phylogenetic distance metric, and NMDS plots to visualize in ordination space. We ran PERMANOVAs with the function *adonis* from the vegan package (Oksanen et al., 2020) to measure the effects of categorical variables and their interactions, and mantel tests for continuous variables. The R package, metacoder (Foster et al., 2017) was used to parse and plot significant ASVs by fluid color based on taxonomy and relative abundance. Some of the Australian *N. mirabilis* samples had lower sequence numbers, so in our comparison of the *N. mirabilis* with *C. follicularis* samples, we rarefied at 1,306 for bacteria and 1,556 for eukaryotes.

For our phylogenetic tree figures, we focused on ASVs that were represented by at least 100 sequences, and that were present in at least 10% of each category for our *Cephalotus*, *Nepenthes*, or *Sarracenia* samples. This is the same criteria used previously (Bittleston et al., 2018) with the goal of avoiding spurious taxa that only randomly or occasionally appeared in pitchers, in order to focus on taxa more likely to be pitcher-associated. These were compared with ASVs from surrounding habitats, including bog water, soil, water held in fallen leaves, and water collected in sterile glass tubes. We used the online interactive tree of life, iToL, software to plot and annotate the trees. The annotations around the outside of the circular trees show the log of the cumulative relative abundance of each ASV, normalized by the number of samples in that category.

Our R code and all the necessary input files and metadata have been deposited in the Harvard Dataverse and are accessible at: https://doi.org/10.7910/DVN/WG1TI6.

## Results

### Characterizing *Cephalotus follicularis*-Associated Communities

#### *Cephalotus follicularis* Pitcher Fluid Volume Varies Across Sites

Despite the proximity of our two sampling sites (roughly 20 km), the volume of pitcher fluid in *C. follicularis* pitchers significantly differed between GRR and TPB samples (Mann–Whitney U test, *Z* = −2.2568, *p* = 0.02402; Supplementary Figure 1A), with higher volumes on average at TPB.

Although there was a trend toward more brown fluid in pitchers at TPB, pitcher fluid color did not differ significantly between sites (Fisher’s exact test of independence, *p* = 0.06006). Pitcher fluid pH and the total extracted DNA concentration from each sample also did not differ by site (Mann–Whitney U tests, *p* > 0.05), and fluid pH also (marginally) did not significantly differ by fluid color (Kruskal-Wallis test, *p* = 0.06763). Fluid pH and DNA concentration were not correlated with volume (linear models, *p* > 0.05); however, fluid pH and DNA concentration were correlated with each other (linear model, *t* value = 2.88, *p* = 0.0065), with more acidic pitchers having lower DNA concentrations (Supplementary Figure 1B). A similar effect of pH on DNA extraction success has been noted in a previous study in *Nepenthes* pitcher plant microbial communities (Gilbert et al., 2020b). A PCA biplot of pitcher pH, volume, and DNA concentration showed separation of the different factors among our samples (Supplementary Figure 1C).

#### Top Organisms in *Cephalotus follicularis*-Associated Communities

We measured community composition of *C. follicularis*-associated bacteria and eukaryotes by analyzing data from 16S and 18S rRNA amplicon sequencing. In the *C. follicularis* 16S sequence data, post-quality control we had 334,718 sequences across 36 samples with a mean of 9,298 sequences per sample and 709 distinct amplicon sequence variants (ASVs). Rarefaction curves showed that all samples reached a plateau in diversity before our rarefaction cutoff (Supplementary Figure 2A). The most abundant phyla were Proteobacteria, Firmicutes, and Bacteroidetes. The taxonomy, relative abundances, and NCBI BLAST results for the top 20 ASVs are reported in Supplementary Dataset 1, accounting for 49.0% of the sequences, and the most abundant families were Burkholderiaceae and Rhodanobacteriaceae, representing 39.7% and 28.0% of the top 20 ASV reads, respectively.

The top bacterial ASV, in the family Burkholderiaceae with the best BLASTn match to *Paraburkholderia aromaticivorans* (Supplementary Dataset 1), had the highest relative abundance of all ASVs at both sampling sites. Interestingly, the second most abundant ASV at each of our sites was in the genus *Rhodanobacter* but was represented by a different ASV at each site. In general, the *C. follicularis* microbiome was dominated by bacteria from the Gammaproteobacteria (Figure 1B).

In the 18S *C. follicularis* sequence data, post-quality control we had 668,485 sequences across 40 samples with a mean of 16,712 sequences per sample and 526 distinct ASVs. Rarefaction curves showed that most samples reached a plateau in diversity before our rarefaction cutoff (Supplementary Figure 2B). The most abundant groups were Metazoa and Fungi, which represented 41.2% and 40.4% of the top 20 ASV reads, respectively (Figure 1B). The taxonomy, relative abundances, and NCBI BLAST results for the top 20 ASVs are reported in Supplementary Dataset 1, accounting for 59.0% of the sequences.

Within Metazoa some of the most commonly identified taxa included: Chromadorea (roundworms), Diptera (flies), Araneae (spiders), and Adinetida (rotifers). The two most common fungal families found were Aspergillaceae and Mortierellaceae.

#### Bacterial and Eukaryotic Richness Are Correlated but Affected by Different Factors

The effective number of species is a measure of alpha diversity or species richness that is calculated as the exponential of the Shannon diversity of species or, as in this case, ASVs. This measure of richness also incorporates the evenness of the community. For bacteria, the effective number of species per sample ranged from 2.5 to 56.7, with a median of 11.1 and a mean of 13.7 (Figure 1C). Eukaryotic communities generally had lower richness with a range of 1.7 to 17, a median of 6 and a mean of 7.1 (Figure 1C). Our results indicate that bacterial and eukaryotic richness are correlated with each other (Table 1; Figure 1D); however, surprisingly, the significant effect is only seen in the bacterial GLM but not the eukaryotic one. This result suggests that the diversity of bacterial taxa increases in response to increased diversity of eukaryotes, although it could also be a response to unmeasured environmental or host factors.

**Table 1:** Factors affecting bacterial and eukaryotic community richness [generalized linear model (GLM) results].

Factor	16S—Bacteria		18S—Eukaryotes	
	*t* Value	Pr(>|*t*|)	*t* Value	Pr(>|*t*|)
(Intercept)	2.845	**0.00854**	1.937	0.0637
pH	−0.522	0.60632	0.353	0.727
Volume	−0.981	0.33561	1.377	0.1802
Site TPB	−1.839	0.07731	−1.08	0.2899
Fluid color, brown	2.14	**0.04194**	1.311	0.2014
Fluid color, cloudy	1.501	0.14531	0.196	0.8464
Fluid color, green	2.772	**0.01016**	0.769	0.449
Fluid color, yellow	1.108	0.27796	−0.646	0.5237
DNA concentration	0.681	0.50173	−2.315	**0.0288**
18S or 16S Effective number of species	2.503	**0.01894**	1.319	0.1985

Bolded values are significant at alpha = 0.05.

Variation in richness in each of the two broad taxonomic groups is associated with different factors. When controlling for other variables, bacterial but not eukaryotic richness significantly varies with color of the pitcher fluid (specifically clear vs. brown and green) but not site, volume or DNA concentration, while the 18S GLM indicates that eukaryotic richness significantly varies only with DNA concentration (Table 1). Effect plots for the bacterial and eukaryotic GLMs are shown in Supplementary Figures 3, 4, respectively.

#### Turnover in Community Composition Varies Significantly by Pitcher Fluid Characteristics, by Site, and Between Bacterial and Eukaryotic Communities

**Figure 2:**
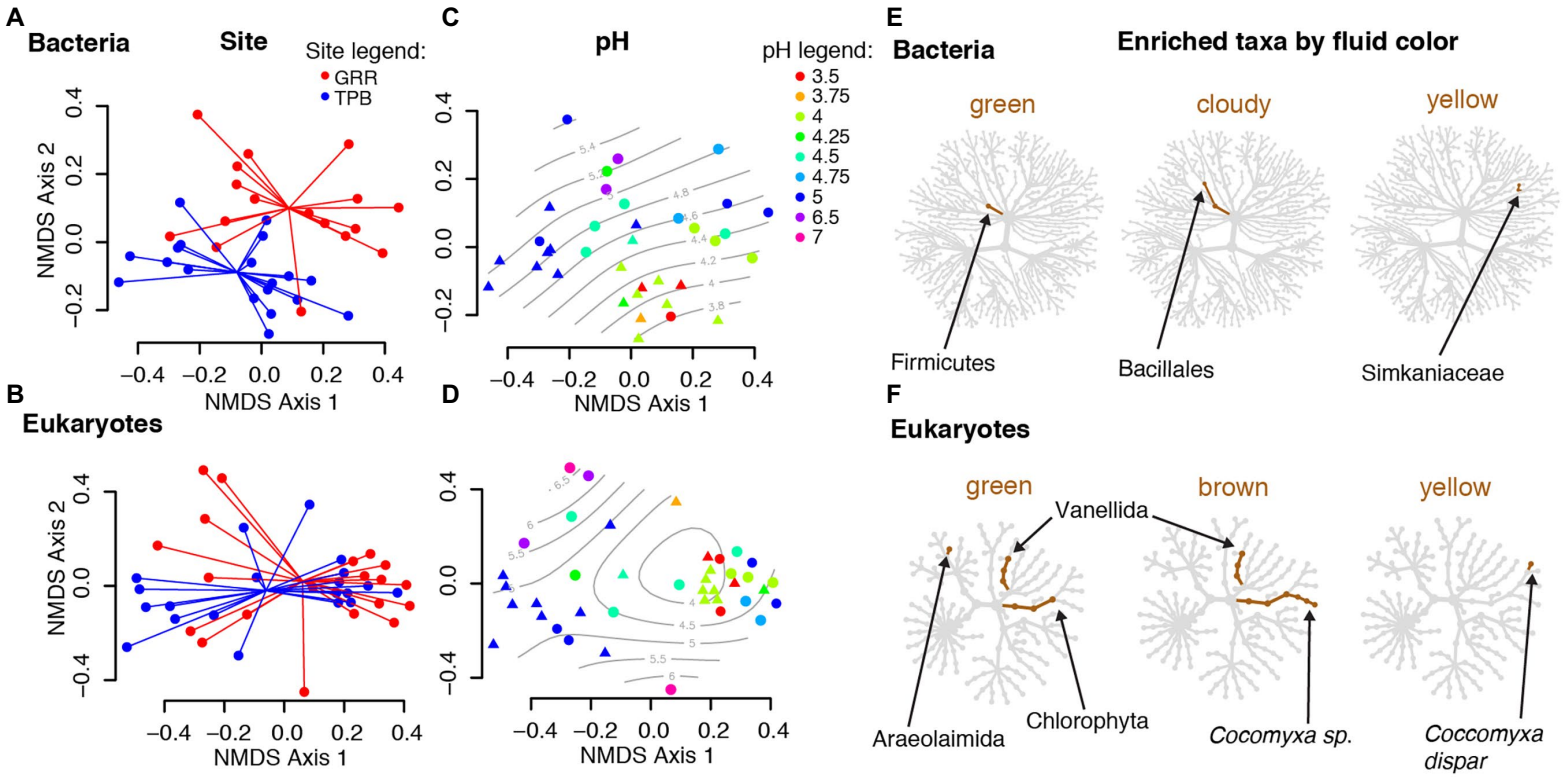
Differences in *Cephalotus follicularis* microbial community composition. **(A–D)** Beta diversity nonmetric multidimensional scaling (NMDS) plots using unweighted UniFrac distances for bacterial and eukaryotic community composition by collecting site and fluid pH. Points in plots **(A)** and **(B)** are colored by site and connected at the mean for each site, while points in plots **(C)** and **(D)** are colored by pH and overlaid with a smooth surface representing pH. **(E,F)** Taxonomic heat trees for bacteria and eukaryotes showing taxa significantly enriched in pitcher fluids of different colors when compared with clear fluid. Taxa shown are enriched by a log_2_ ratio median proportion of 3.

Despite our two sites being less than 20 km apart, both pitcher bacterial and eukaryotic communities show significant differences in compositional turnover (beta diversity) by site, which explains ~4–5% of the existing variation (PERMANOVA, Bacteria *R*^2^ = 0.04215, *p* = 0.010; Eukaryotes *R*^2^ = 0.04755, *p* = 0.001; Table 2). Our NMDS plots indicate separation by site diagonally along both axes 1 and 2 for bacterial and eukaryotic communities (Figures 2A,B). For both bacterial and eukaryotic community compositions, a large portion of the variation is explained by fluid pH (mantel tests: Bacteria *r* = 0.2837, *p* = 0.001; Eukaryotes *r* = 0.3903, *p* = 0.001; Table 2; Figures 2C,D). In the NMDS plots, bacterial communities have more distinct clustering by site, and a more linear gradient of community composition dissimilarity by fluid acidity, than eukaryotic communities. Although exact reasons for the color change of pitcher fluids are not fully known, fluid color is also correlated with specific pitcher communities, and explains more of the variation in community composition than site does for both bacteria and eukaryotes (Bacteria *R*^2^ = 0.16485, *p* = 0.001; Eukaryotes *R*^2^ = 0.12727, *p* = 0.001). In eukaryotes, there is a significant interaction between site and fluid color (*R*^2^ = 0.09836, *p* = 0.007), while in bacteria, the interaction shows a trend that is not significant (*R*^2^ = 0.08946, *p* = 0.076). Community compositions of bacteria and eukaryotes are both not significantly correlated with pitcher fluid volume, and, similar to species richness (alpha diversity), only eukaryotic community composition (beta diversity) varies significantly with DNA concentration (Table 2). The strongest beta diversity correlation is seen between the weighted UniFrac dissimilarities of bacteria and eukaryotes themselves (mantel test, *r* = 0.4026, *p* = 0.001; Table 2).

**Table 2:** Factors affecting bacterial and eukaryotic turnover in community composition.

Factor	Bacteria—16S		Eukaryotes—18S	
**PERMANOVA**	***R*** ^**2**^	***p***	***R*** ^**2**^	***p***
Fluid color	**0.16485**	**0.001**	**0.12727**	**0.006**
Site	**0.04215**	**0.010**	**0.04755**	**0.001**
Fluid color:site	0.08946	0.076	**0.09836**	**0.007**
**Mantel tests**	***r***	***p***	***r***	***p***
pH	**0.2837**	**0.001**	**0.3903**	**0.001**
Volume	0.03139	0.315	−0.07258	0.891
DNA concentration	0.08681	0.125	**0.2853**	**0.001**
18S	**0.4026**	**0.001**		

Bolded values are significant at alpha = 0.05.

#### Specific Taxa Are Present in *Cephalotus follicularis* Pitcher Fluids of Different Colors

Our taxonomic heat tree analysis found no bacterial taxa that significantly differed by site, however there were bacterial taxa with significant differences based on pitcher fluid color: taxa in the phylum Firmicutes were enriched in green fluids, taxa in the order Bacillales were enriched in cloudy fluids and taxa in the family Simkaniaceae (order Chlamydiales) were enriched in yellow fluids (Figure 2E).

Analysis of the eukaryotic data found one significantly differing taxon by site, a fungus classified as *Mortierella* (phylum Mucoromycota) enriched at the TPB site. As with bacteria, eukaryotic taxa—specifically different groups of green algae—significantly differed with the color of the pitcher fluid: Chlorophyta were generally enriched in green fluid, Chlorophyta plus a *Cocomyxa* species in brown fluid and *Cocomyxa dispar* in yellow fluid. In addition to the green algae, Vannellida (an order of freshwater and marine amoebae) was enriched in both green and brown fluids, and Araeolaimida (freshwater nematodes) were enriched in green fluids (Figure 2F).

### Comparing Communities Across Independently Evolved Hosts

#### In a Global Comparison, *Cephalotus follicularis* Communities Converge With Those of *Nepenthes* and *Sarracenia*

**Figure 3:**
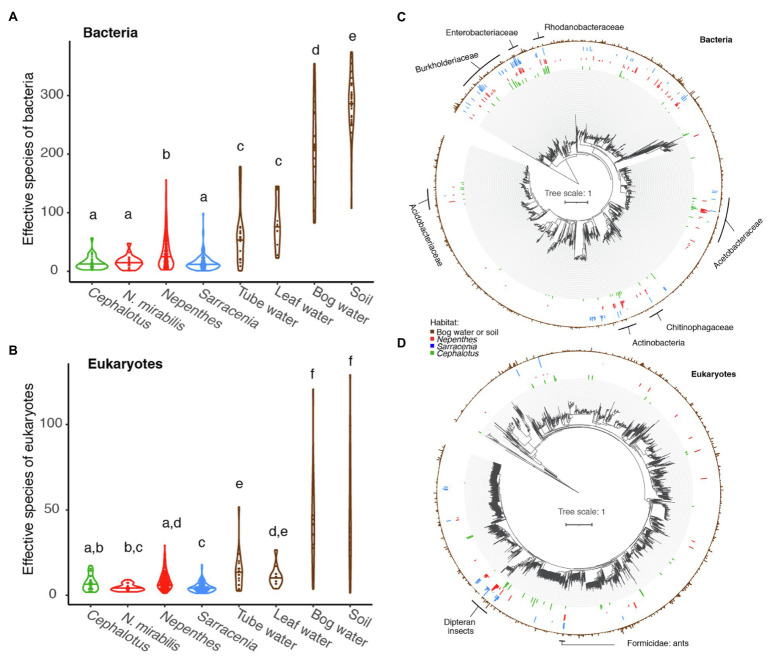
Global comparison of pitcher plant-associated communities. **(A,B)** Richness measured as effective number of species for *Cephalotus follicularis* bacteria **(A)** and eukaryotes **(B)** in comparison with Australian *Nepenthes mirabilis*, other *Nepenthes*, *Sarracenia*, and environmental communities. Different letters above the violin plots represent significant differences as measured by a pairwise Wilcoxon test, with Benjamini and Hochberg adjustment for multiple comparisons. **(C,D)** Phylogenetic trees of bacteria **(C)** and eukaryotes **(D)**, comparing organisms from environmental samples (brown, outer ring) with those found in at least 10% of our *Cephalotus follicularis* (green, innermost ring), *Nepenthes* (red, second innermost ring) and *Sarracenia* (blue, second outermost ring) samples. Names of selected shared clades are listed.

The alpha diversity, or richness as measured by effective number of species, of *C. follicularis* pitcher communities is similar to that of *Nepenthes* pitcher communities in Australia and Southeast Asia and *Sarracenia* pitcher communities in North America. All pitcher communities are relatively low compared with communities of water-filled fallen leaves and other environmental samples collected from surrounding habitats (Figures 3A,B). This is true for both bacteria and eukaryotes, with a few slight differences. *Cephalotus follicularis* bacterial richness is not significantly different when compared with Australian *N. mirabilis* or North American *Sarracenia* bacterial richness but is significantly lower than non-Australian *Nepenthes*, and much lower than all environmental samples (Figure 3A). *Cephalotus follicularis* eukaryotic richness is not significantly different when compared with Australian *N. mirabilis* or other *Nepenthes* but is significantly higher than *Sarracenia*, and much lower than all environmental samples (Figure 3B).

The bacteria in *C. follicularis* pitchers tend to come from the same phylogenetic clades as those from *Nepenthes* and *Sarracenia* pitchers (Figure 3C). Clades with shared taxa among all three pitcher plant genera include: Burkhoderiaceae, Enterobacteriaceae, Rhodanobacteraceae, Acetobacteraceae, Chitinophagaceae, Actinobacteria, and Acidobacteriaceae. In contrast, many fewer eukaryotic taxa are shared with *Nepenthes* and *Sarracenia* with one exception: flies in the order Diptera (flies, mosquitos, midges, etc.) show a clear, strong signal of high relative abundance across all three pitcher plant genera (Figure 3D) and another shared group maps to Formicidae (ants).

#### Similarities and Differences of *Cephalotus follicularis* Communities vs. Australian *Nepenthes mirabilis*

Our rationale for selecting and presenting a comparison sample is largely heuristic. The closest pitcher plants to *C. follicularis* are on the opposite side of the continent. Since detailed and repeated sampling over time was not possible, for this initial, descriptive comparison of the pitcher microcosms from *C. follicularis* with those of other pitcher plant species, we identified a similarly sized, naturally occurring population of *N. mirabilis* pitcher plants in Northeast Australia, and sampled them at a time where pitchers were actively being produced in a manner similar to those observed in the *C. follicularis* population. Although the calendar dates for comparison sampling both fell within 2016, they were separated by 5 months (January for *C. follicularis*, roughly midsummer at a temperate, less humid latitude, and May for *N. mirabilis*, the start of winter months at a humid, tropical latitude on the opposite side of the country, more than 3,400 km away).

The 21 samples collected from *N. mirabilis* at one site near Bramston Beach hosted communities similar to those of *C. follicularis* with regard to bacterial taxa, with Gammaproteobacteria and Alphaproteobacteria at similar relative abundances and the highest representation. However, there were relatively more taxa in the class Bacteroidia in *N. mirabilis*, and fewer in the classes Actinobacteria and Acidobacteriia (Supplementary Figure 5). The two Australian pitcher plant species were more different with regard to their associated eukaryotic taxa; in particular *N. mirabilis* had relatively more Metazoa (mostly represented by insects) and fewer Fungi (Supplementary Figure 5). In terms of species richness, the Australian *N. mirabilis*-associated had fewer eukaryotic ASVs, on average, than the Southeast Asian *Nepenthes* communities (Figure 3B).

## Discussion

The pitchers of *C. follicularis* represent a unique habitat that is vulnerable to extinction along with its charismatic host plant (Conran, 2004). Here, for the first time, we used metabarcoding to characterize the full eukaryotic and bacterial communities living within *C. follicularis* pitchers and compared them with pitcher communities from across the globe. We found differences in the factors affecting richness of *C. follicularis* bacterial and eukaryotic communities (Table 1), while factors affecting community composition were generally more similar (Table 2; Figure 2). We also saw convergence in the richness and composition of bacterial taxa of *C. follicularis* when comparing with the convergently evolved *Nepenthes* and *Sarracenia* species from other parts of the world (Figure 3). Eukaryotic taxa showed less striking convergence, with strong similarities in richness but primarily only dipteran insects as shared inquilines. In our samples, *C. follicularis* pitchers tended to have fewer arthropod inquilines than have been reported from *Nepenthes* and *Sarracenia* pitchers. The consistently small *C. follicularis* pitcher size may be partly responsible for the differences in eukaryotic taxa, but it also seems possible that their highly isolated and remote location in Western Australia—almost an island within an island—may have limited the kinds and numbers of invertebrate colonists the pitchers have received (Beaver, 1985; Clarke, 1998).

Previous studies have noted differences in factors affecting the diversity of bacterial and eukaryotic communities, as well as correlations between these different taxa (Ragon et al., 2012; Ramirez et al., 2014; Li et al., 2017). For example, a study of *Nepenthes*-associated communities along an elevational gradient in the Philippines found clear differences in the environmental and host factors affecting bacterial and eukaryotic communities, with elevation having a stronger effect on eukaryotes while pH had a stronger effect on bacteria (Gilbert et al., 2020a). Another study across the communities of eight *Nepenthes* species found a strong correlation between bacterial and eukaryotic community composition, and noted tight clustering of protist and bacterial taxa in a co-occurrence network (Bittleston, 2018). Correlations in the diversity of eukaryotic and bacterial communities could be driven by species interactions; for example, a more diverse suite of eukaryotic algae may lead to a more diverse suite of bacteria able to metabolize a variety of algal compounds.

When exploring the richness of *C. follicularis* pitcher-associated communities, we found that bacterial and eukaryotic richness were correlated, but potentially driven by different factors. Bacterial richness differed by the color of the pitcher fluid, while eukaryotic richness differed by DNA concentration. It is unclear why eukaryotic richness would decrease with increasing DNA concentration, since DNA concentration can be a proxy for biomass within the sample. However, it could indicate a bloom of a particular organism: if one species multiplied quickly, it would increase biomass of the focal species while decreasing the overall effective number of species. We also noted a weak correlation between DNA concentration and fluid pH, which either could suggest that lower pH environments house fewer organisms and thus have lower overall biomass, or could suggest that DNA is more difficult to extract from lower pH fluids. In soils, lower pH is associated with decreased microbial growth rates but not biomass (Malik et al., 2018) and can cause adsorption of extracellular DNA to adsorption onto the soil matrix (Young et al., 2014). It is unclear if these processes would apply to pitcher fluid.

In contrast to our richness results, the turnover in community composition of bacterial and eukaryotic communities was generally affected by the same factors, with few exceptions. Both pH and fluid color explained relatively large portions of the variation in bacterial and eukaryotic communities, and the site was also significant although with smaller effect sizes, while neither taxonomic group was significantly explained by pitcher fluid volume. As with richness, only eukaryotic community composition had a significant relationship with DNA concentration. The largest effect size from our mantel tests was for the correlation between bacterial and eukaryotic phylogenetic dissimilarities themselves, suggesting that bacterial and eukaryotic compositions change across samples in similar ways, either due to species interactions or to similar responses to habitat differences.

Our analysis using taxonomic heat trees revealed enrichment of certain taxa in different colors of pitcher fluid and one between sites. The taxa in differently colored fluids likely either caused the color changes or were affected by a shift in chemistry or nutrients related to a color change. Green, brown, and yellow fluids were enriched with algae in the Chlorophyta group, while cloudy fluids were enriched with bacteria in the order Bacillales. The algae likely contributed to the green, brown, and yellow coloration, while the bacteria may have contributed to the cloudiness. Only one taxon differed significantly by site, the fungal genus *Mortierella*. Species of *Mortierella* are generally decomposers, and some have the ability to degrade the chitin in insect exoskeletons (Okafor, 1966; Edgington et al., 2014) or to enhance plant growth when part of the rhizosphere (Li et al., 2020). Potentially the *Mortierella* that were enriched at the TPB site were involved in breaking down insect prey captured by *C. follicularis* pitchers, and a possible symbiosis between the fungus and pitcher plant would be interesting to explore further.

Pitcher communities change over successional time, from pitcher opening to senescence. This successional change has been well-documented only in *Sarracenia purpurea* (e.g., Gray, 2012; Miller and terHorst, 2012; Luciano and Newell, 2017; Boynton et al., 2019), but occurs in *N. mirabilis* (Benson, Bittleston, and Pierce, unpublished results), and likely also in *C. follicularis*. Limitations of our current study prevented us from sampling the same pitchers at different successional stages; however, we aimed to select healthy pitchers of different ages to get a full representation of the existing diversity. Future studies in *C. follicularis* could sample across different seasons and successional stages.

All three genera of pitcher plants host specialized insects (Adlassnig et al., 2011), and host specificity of inquilines could lead to co-evolution of the plant and certain associated organisms over time. For example, in the *Sarracenia alata* system, pitcher communities—and arthropods in particular—have co-diversified with their host plants across a biogeographic barrier (Koopman and Carstens, 2011; Satler and Carstens, 2016, 2019). The main eukaryotic taxonomic group showing convergence in our study is Diptera, or flies. These highly-represented ASVs likely come from DNA of specialized inquilines and not from prey, since previous pitcher plant studies have demonstrated corroboration between amplicon sequencing-derived taxa and barcoded DNA extracted directly from inquilines (Bittleston et al., 2016a). The most abundant dipteran ASVs from *C. follicularis* map most closely to *Drosophila*; however, it is likely that they are actually the inquiline fly *Badisis ambulans*, whose larvae we saw swimming in our pitcher samples. *Badisis ambulans* is not represented in the NCBI database, in fact, currently only one taxon in the whole Micropezidae family is represented by a section of the 18S rRNA gene sequence. The dipteran ASVs from *Nepenthes* and *Sarracenia* are generally assigned to the mosquitoes, midges, and other flies that are known inquilines of these plants (Bittleston et al., 2016a, 2018). Overall, we saw lower eukaryotic species richness in the Australian *N. mirabilis* pitchers when compared with other *Nepenthes*, potentially because Australian *N. mirabilis* is geographically isolated on the edge of the *Nepenthes* distribution without other co-occurring *Nepenthes* species (Beaver, 1985; Clarke, 1998). Similarly, *C. follicularis* had fewer insect ASVs than *N. mirabilis* and the other pitcher plant species, perhaps due to a combination of geographic isolation and small size. The other eukaryotic group showing convergence across pitcher plant genera was Formicidae, or ants, and these ASVs likely come from the primary prey of pitcher plants, as all three groups commonly capture and digest ants (Ellison and Gotelli, 2009).

Beyond arthropods, many bacterial groups were present across the three genera of pitcher plants. It is possible that taxa in these shared groups (such as the Acetobacteraceae, Rhodanobacteraceae, Chitinophagaceae, Burkholderiaceae, among others) could be specialists in the pitcher habitats; however, host specificity in bacteria is rare and difficult to test. It is more likely that they are present due to environmental filtering as a result of host convergence with regard to the pitcher habitat. Pitchers are not just passive receptacles, they affect the habitat inside *via* excretion of digestive enzymes, oxygenation, and sometimes active pH adjustment (Bradshaw and Creelman, 1984; An et al., 2001; Adlassnig et al., 2011; Sirota et al., 2013; Ravee et al., 2018; Gilbert et al., 2020b). A recent experiment found *S. purpurea* regulates the structure of its inquiline food web, and direct contact with the plant tissue was important (Ellison et al., 2021). Similarities in the chemistry and conditions of other, non-host associated habitats can also lead to convergence in bacterial communities; for example, similarities in pH, salinity and other soil characteristics drive similarity in soil microbial communities (Ramirez et al., 2014; Rath et al., 2019). However, non-host associated systems are less likely to represent convergent interactions (Bittleston et al., 2016b), where a specific functional interaction—such as bacteria assisting pitcher plants in the degradation of insect prey—emerges independently in multiple instances. Due to the combination of convergent evolution at the host level and likely chemical similarities at the pitcher habitat level, we consider this convergence as both evolutionary and ecological.

The similarities in richness and bacterial taxa in *C. follicularis*, *Nepenthes* and *Sarracenia* pitcher communities in very different parts of the world—Australia, Southeast Asia and North America—suggests that pitchers in general cultivate a particular habitat that selects for a similar microbiome, regardless of location or evolutionary history (Bittleston et al., 2018). This is not to say that communities hosted by different genera of pitcher plants do not show variation. In fact, each particular plant can host unique combinations of taxa that, at the fine scale, vary between genus, species, site, and even within a population due to unique characteristics like pitcher fluid acidity. However, at a broad scale, pitcher microbial communities on opposite sides of the world are more similar to each other than to the water-filled leaf, bog water, or soil communities in the surrounding habitats.

## Data Availability Statement

The names of the repositories and accession numbers are: https://www.ncbi.nlm.nih.gov/bioproject/PRJNA810039; https://doi.org/10.7910/DVN/WG1TI6.

## Author Contributions

LB and NP conceived of the study. LB and EB collected and processed samples. LB, EB, and JB analyzed the data. All authors helped to write and edit the manuscript. All authors contributed to the article and approved the submitted version.

## Funding

The collection of *Cephalotus follicularis* samples was funded by a Putnam Expedition Grant from the Harvard Museum of Comparative Zoology (MCZ) to LB. The collection of *Nepenthes mirabilis* samples was funded by a Bolitho Scholarship from the Harvard University Committee on Australian Studies. Partial funding for the DNA extraction and amplification and initial analyses was provided by The Museum of Comparative Zoology, Harvard University Herbaria, and Arnold Arboretum Grant-in-aid of Undergraduate Research and Harvard College Research Program Undergraduate Summer Funding to EB. LB and JB are currently funded by an NSF award 2025250, and LB is also supported by the NSF Idaho EPSCoR Program and award number OIA-1757324. Published by a grant from the Wetmore Colles Fund.

## Conflict of Interest

The authors declare that the research was conducted in the absence of any commercial or financial relationships that could be construed as a potential conflict of interest.

## Publisher’s Note

All claims expressed in this article are solely those of the authors and do not necessarily represent those of their affiliated organizations, or those of the publisher, the editors and the reviewers. Any product that may be evaluated in this article, or claim that may be made by its manufacturer, is not guaranteed or endorsed by the publisher.
