# Supplementary material for: Characterization and Comparison of Convergence Among Cephalotus follicularis Pitcher Plant-Associated Communities With Those of Nepenthes and Sarracenia Found Worldwide
Source: Front Plant Sci. 2022 Jun 6;13:887635. doi: 10.3389/fpls.2022.887635 (PMC9207445; doi:10.3389/fpls.2022.887635)
Supplement: Supplementary file 2 [file Data_Sheet_1.docx]

**Supplementary Figures**


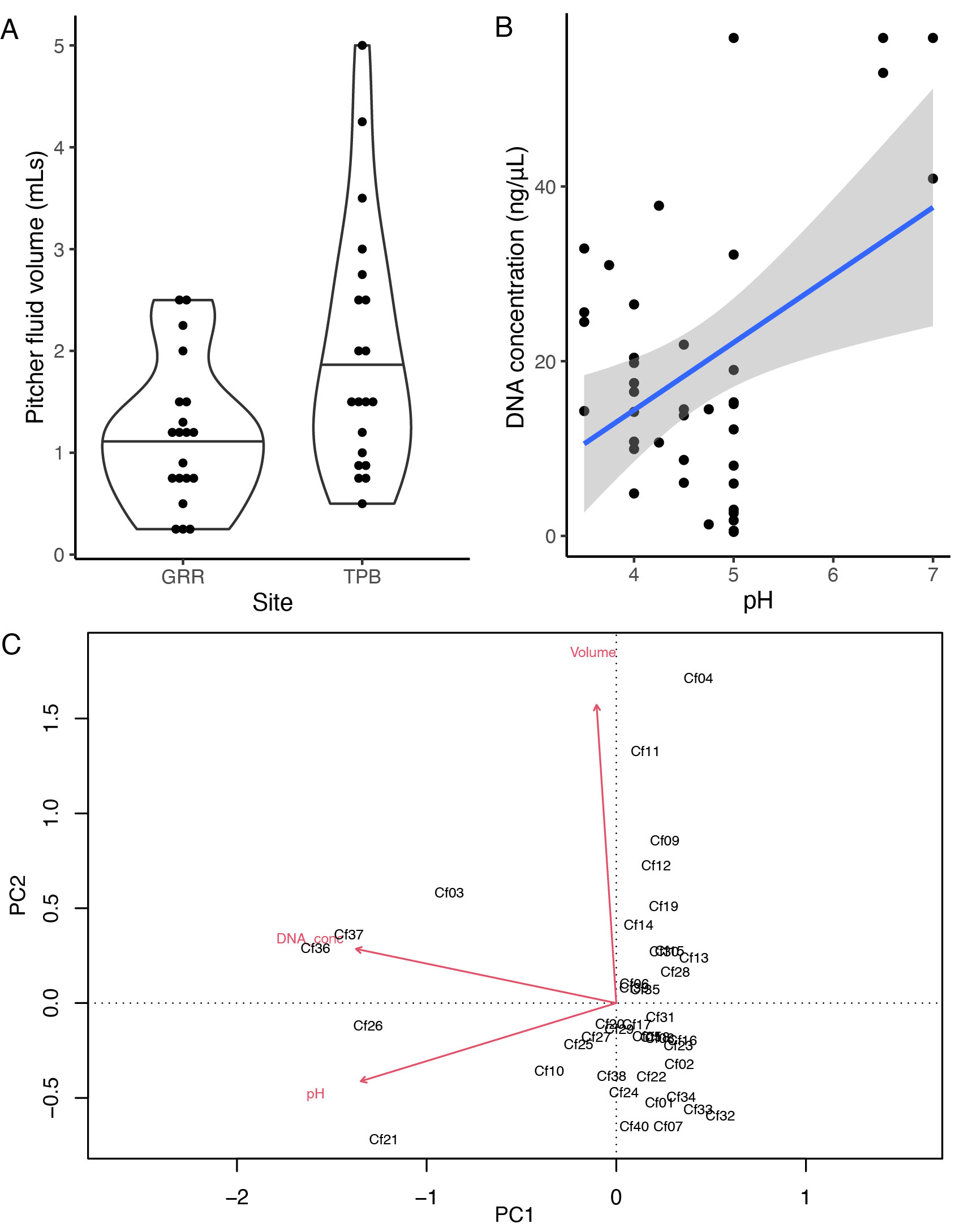


**Supplementary Figure 1**. Comparing environmental variables for *C. follicularis* samples. A) A violin plot showing significant differences in pitcher fluid volume by site. B) Scatterplot showing the significant correlation between pitcher fluid pH and the concentration of DNA extracted from the sample. C) Principle Component Analysis (PCA) biplot of pitcher fluid pH, volume, and DNA concentration with the factors mapped onto the ordination.

**
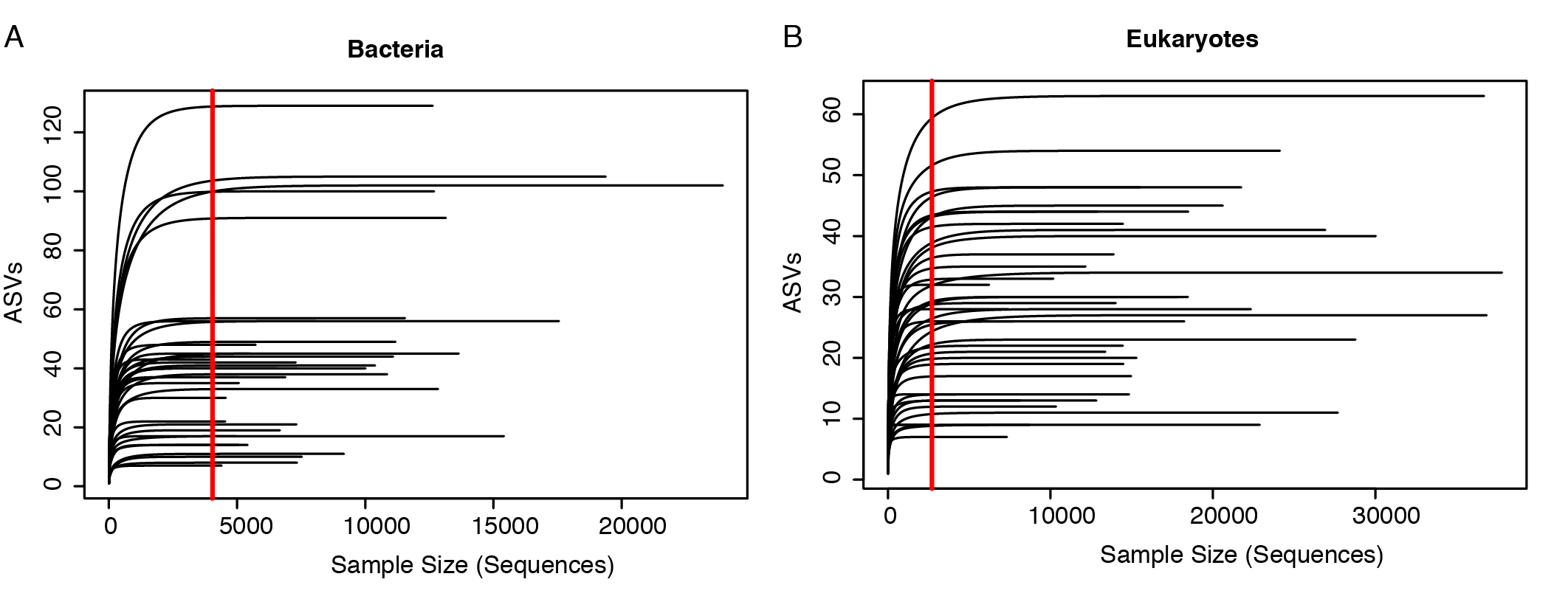
**

**Supplementary Figure 2.** Rarefaction curves for *Cephalotus follicularis* amplicon sequencing for A) bacteria (16S) and B) eukaryotes (18S). Rarefaction cutoffs for each dataset are shown as red lines.

**Supplementary Figure 3.** Effect plots for the 16S alpha diversity Gamma distributed Generalized Linear Model (GLM)

**Supplementary Figure 4.** Effect plots for the 18S alpha diversity Gamma distributed Generalized Linear Model (GLM)

**
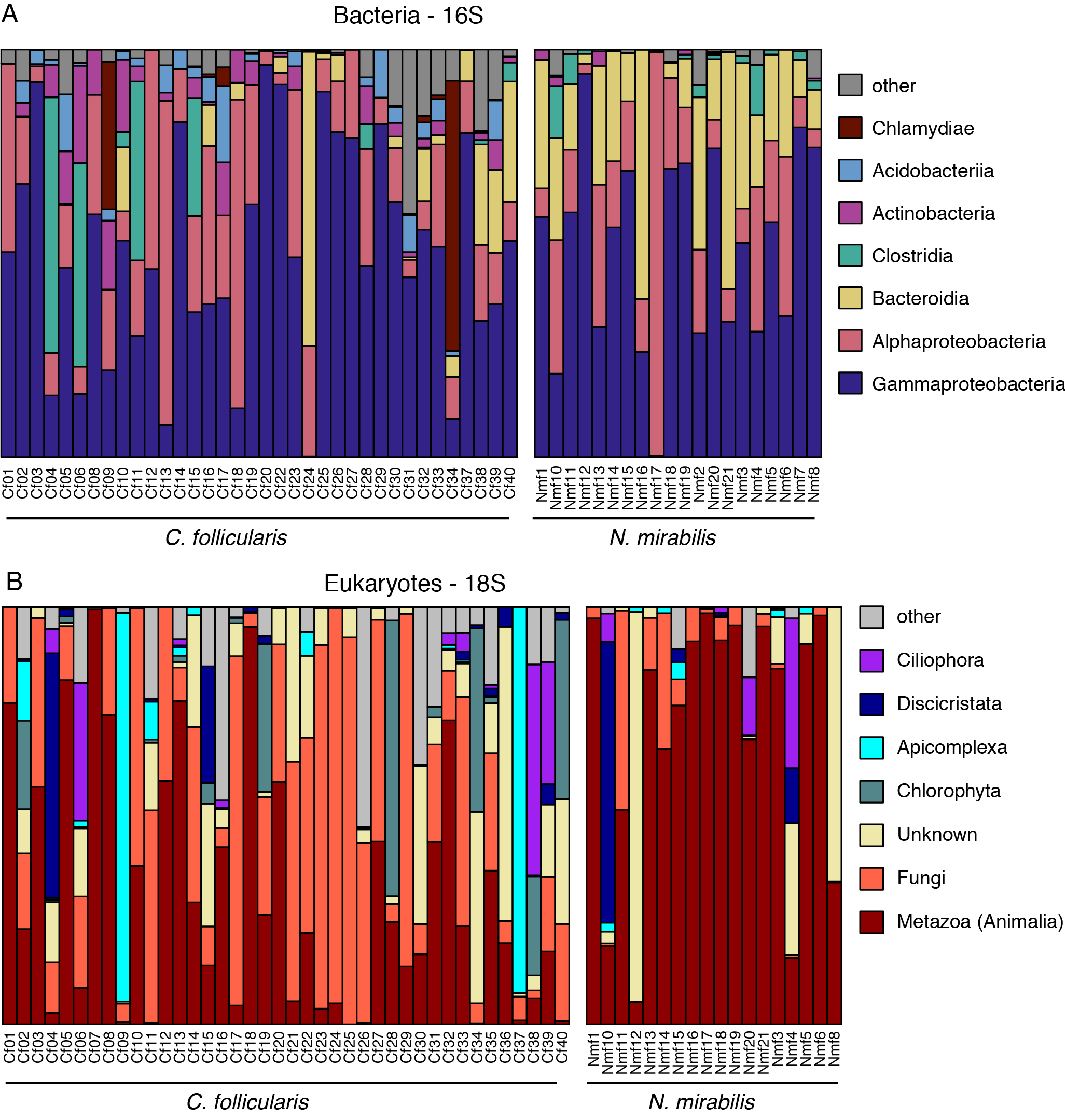
**

**Supplementary Figure 5**. Comparison of the relative abundance of A) bacterial and B) eukaryotic taxa in Australian *C. follicularis* and *N. mirabilis* pitchers
